# Low‐Cost Preparation of High‐Performance Na‐B‐H‐S Electrolyte for All‐Solid‐State Sodium‐Ion Batteries

**DOI:** 10.1002/advs.202302618

**Published:** 2023-09-25

**Authors:** Wei Zhou, Changsheng Song, Shuyang Li, Miao Liu, Huiwen He, Shaoyu Yang, Jin Xie, Fei Wang, Fang Fang, Dalin Sun, Jie Zhao, Yun Song

**Affiliations:** ^1^ Department of Materials Science Fudan University Shanghai 200433 China; ^2^ State Key Laboratory of Molecular Engineering of Polymers Department of Material Science Fudan University Shanghai 200438 China; ^3^ Beijing National Laboratory for Condensed Matter Physics and Institute of Physics Chinese Academy of Sciences Beijing 100190 China; ^4^ State Key Laboratory of Power Grid Environmental Protection China Electric Power Research Institute Wuhan 430074 China; ^5^ School of Physical Science and Technology ShanghaiTech University Shanghai 201210 China

**Keywords:** all‐solid‐state batteries, low‐cost, solid electrolytes, superionic conductors, thioborate

## Abstract

All‐solid‐state sodium‐ion batteries have the potential to improve safety and mitigate the cost bottlenecks of the current lithium‐ion battery system if a high‐performance electrolyte with cost advantages can be easily synthesized. In this study, a one‐step dehydrogenation‐assisted strategy to synthesize the novel thio‐borohydride (Na‐B‐H‐S) electrolyte is proposed, in which both raw material cost and preparation temperature are significantly reduced. By using sodium borohydride (NaBH_4_) instead of B as a starting material, B atoms can be readily released from NaBH_4_ with much less energy and thus became more available to generate thio‐borohydride. The synthesized Na‐B‐H‐S (NaBH_4_/Na‐B‐S) electrolyte exhibits excellent compatibility with current cathode materials, including FeF_3_ (1.0–4.5 V), Na_3_V_2_(PO_4_)_3_ (2.0–4.0 V), and S (1.2–2.8 V). This novel Na‐B‐H‐S electrolyte will take a place in mainstream electrolytes because of its advantages in preparation, cost, and compatibility with various cathode materials.

## Graphical abstract

The novel thio‐borohydride (Na‐B‐H‐S) electrolyte is prepared by a one‐step dehydrogenation‐assisted strategy. By using NaBH_4_ instead of B as a starting material, B atoms are released from NaBH_4_ with much less energy and thus became more available to generate thio‐borohydride. The synthesized Na‐B‐H‐S (NaBH_4_/Na‐B‐S) electrolyte exhibits excellent compatibility with current cathode materials, including FeF_3_, Na_3_V_2_(PO_4_)_3_, and S.

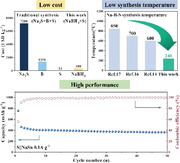

## 1 Introduction

To assuage recent concerns about the safety of lithium and lithium resource shortage issues, sodium‐ion batteries have emerged as promising alternative candidates.^[^ ^1^, ^2^ ^]^ To mitigate recent concerns about the safety of lithium as well as lithium resource shortage issues, all‐solid‐state sodium‐ion batteries have emerged as promising alternative candidates, continuously promoting research on fast sodium ionic electrolytes.^[^ ^3^, ^4^, ^5^, ^6^, ^7^, ^8^ ^]^ Among these electrolytes, NaBH_4_‐based electrolytes have attracted growing research attention because of their light weight, low cost, and simple device integration.^[^ ^9^, ^10^ ^]^ Matsuo et al.^[^ ^11^ ^]^ first investigated the Na^+^ conductivity of pure NaBH_4_ and showed that it had a value of 10^−10^ S cm^−1^ at room temperature, although it was still far from achieving the available target of 10^−4^ S cm^−1^.

As an inexpensive industrial reducing agent, the significant cost advantage of NaBH_4_ has inspired interest in improving its Na^+^ conductivity. The most straightforward way to improve conductivity is using a second‐phase incorporation strategy, such as NaBH_4_‐C_60_,^[^ ^12^ ^]^ NaBH_4_‐Al_2_O_3_,^[^ ^13^ ^]^ or NaBH_4_‐SiO_2_.^[^ ^13^ ^]^ The NaBH_4_‐C_60_ nanocomposite has shown an increase of four orders of magnitude in ionic conductivity compared with pure NaBH_4_. More recently, the NaBH_4_‐Al_2_O_3_ nanocomposite exhibited an ionic conductivity of 7.2 × 10^−6^ S cm^−1^ at 80 °C. These second phases were incorporated through the ball‐milling method or the melt infiltration process; in other words, two phases were mixed using a physical approach. Ngene et al.^[^ ^13^ ^]^ revealed that this second‐phase strategy could be attributed to the surface interaction between the oxides (Al_2_O_3_ and SiO_2_) and NaBH_4_. To strengthen the two‐phase interface, the Na_2_B_12_H_12_ phase was generated in situ through a gas‐solid chemical reaction between NaBH_4_ and B_2_H_6_. The end product of the NaBH_4_‐Na_2_B_12_H_12_ composite achieved a sodium ionic conductivity of 10^−4^ S cm^−1^ at 115 °C.^[^ ^9^ ^]^

The phase structure and incorporation method both played key roles in tuning the Na^+^ conductivity of NaBH_4_. Enlighted by the finding that an increase of three orders of magnitude in ionic conductivity could be obtained by simple substitution of O in Li_3+x_(P_1‐x_Si_x_)O_4_ with S,^[^ ^14^, ^15^, ^16^ ^]^ we speculated that introducing Na‐B‐S (e.g., NaBS_3_, Na_2_B_2_S_5_, and Na_3_B_3_S_6_) as the second phase to fabricate thio‐borohydride composites could lead to achievements of novel properties and extend new chemistry in the NaBH_4_ electrolyte. The following consequential issues, however, are related to the sodium thioborate system (Na‐B‐S): i) high energy consumption, the raw material B_2_S_3_ is not commercially available, high temperatures ranged from 600–850 °C is needed to ensure the reaction between boron and sulfur powder;^[^ ^17^, ^18^, ^19^ ^]^ ii) the synthesized B_2_S_3_ is unstable upon multi‐step reactions due to its low chemical stability;^[^ ^18^ ^]^ iii) raw material costs also need to be considered.

Herein, thioborate (Na‐B‐S) was generated in situ within NaBH_4_ through chemical reactions between NaBH_4_ and S, which are both low‐cost raw materials. Additionally, with the aid of the partial dehydrogenation of NaBH_4_, the synthesized temperature could be significantly decreased to 240 °C. Such a strategy of incorporating second‐phase Na‐B‐S through an in situ chemical reaction undoubtedly produces the effect of killing two birds with one stone, the cheapest stone. The synthesized NaBH_4_/Na‐B‐S (Na‐B‐H‐S) electrolyte exhibited a Na^+^ conductivity of 1.7 × 10^−4^ S cm^−1^ at 120 °C, which was an increase of three orders of magnitude compared with pristine NaBH_4_. When assembled with the S cathode and Na_15_Sn_4_ anode, the S||Na_15_Sn_4_ device achieved a capacity of 364 mAh g^−1^ after 50 cycles. Notably, a capacity of 78 mAh g^−1^ was obtained in the Na_3_V_2_(PO_4_)_3_||Na_15_Sn_4_ device after 50 cycles. Such low‐cost and low‐energy‐consumption preparation of the novel Na‐B‐H‐S electrolyte, along with success in assembling Na_3_V_2_(PO_4_)_3_ cathode, will shed light on further commercialization.

## 2 Results and Discussion

**Figure 1:**
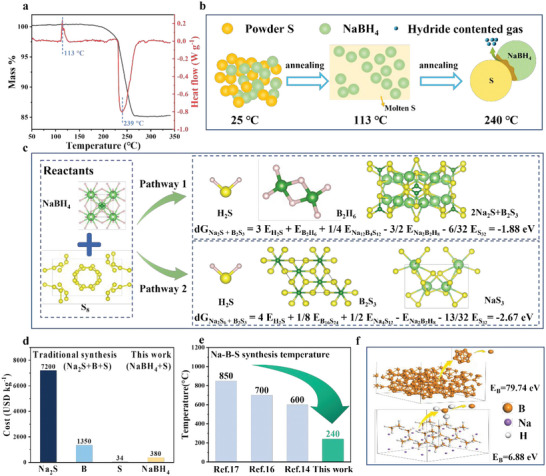
Synthesis of Na‐B‐H‐S electrolytes. a) TG‐DSC curve for as‐milled NaBH_4_‐S. b) The schematic view of the reaction process between NaBH_4_ and S during the annealing. c) The Gibbs energy of the Gibbs energy of two kinds of formation process by DFT simulation. d) The cost of raw materials of B (95%), Na_2_S (95%), S (99.5%), and NaBH_4_ (98%) (excerpted from Aladdin, https://www.aladdin‐e.com/zh_cn/). e) The Na‐B‐S reaction temperature comparison in references and this work. f). Comparison of the energy (*E*_a_, in eV) required to remove one B atom from the surfaces of crystalline B and NaBH_4_.

We employed TG‐DSC to determine the thermodynamic properties between NaBH_4_ and S, as shown in **Figure** **1**a. The minor endotherm peak could be found ≈113 °C without weight loss because of the polymorphic transition and subsequent melting of S.^[^ ^20^, ^21^, ^22^ ^]^ The reaction between NaBH_4_ and S occurred rapidly at 239 °C, as represented by the sharp exotherm peak. Corresponding to this peak, the TG curve shows a significant weight loss at temperatures between 200 and 260 °C. As schematized in Figure1b, S initially underwent a melting process at 113 °C to form the mixture of NaBH_4_ and molten S. Once the temperature increased to 239 °C, the molten S was reacted with partially dehydrogenated NaBH_4_. Note that the pure NaBH_4_ released hydrogen higher than 400 °C, and the existence of molten S might have catalyzed the partial dehydrogenation of NaBH_4_ at 239 °C.^[^ ^23^ ^]^ To further estimate the feasibility of this reaction, we employed a theoretical simulation, as shown in Figure 1c. First, obtained from Atomly, the open‐access density functional theory materials data infrastructure,^[^ ^24^ ^]^ the existing stable Na‐S phases were Na_2_S, NaS, NaS_2_, Na_2_S_5_, and NaS_3_, as illustrated in Figure S1 (Supporting Information). The highest and lowest Na/S ratios (Na_2_S and NaS_3_) were assumed to be the end product of the reaction between NaBH_4_ and S, as labeled in reactions one and two, respectively. The overall free energy of reactions one and two was calculated to be −2.67 eV and −1.88 eV, respectively. These two calculated negative free energy changes demonstrated the feasibility of the reaction between NaBH_4_ and S, even with a varied and uncertain Na‐S end product.

To highlight the cost advantage of the NaBH_4_+S reaction route, we compared various raw materials (B, >95%; Na_2_S, >95%; S, >99.5%; NaBH_4_, >98%), as shown in Figure 1d (excerpted from the website of Aladdin). In the traditional synthesis route, Na_2_S provided both Na and S sources, and B powder was the B source, whereas S powder was the additional S source.^[^ ^17^, ^18^, ^19^ ^]^ In contrast, raw materials were simplified in the NaBH_4_+S reaction route. The NaBH_4_ provided both Na and B sources, whereas S powder was the S source, in which the cost of raw material could be sharply decreased because both NaBH_4_ and S were industrial materials. For the traditional route, the ultra‐high price of Na_2_S could be attributed to the time‐ and energy‐intensive process necessary to obtain anhydrous Na_2_S.^[^ ^25^ ^]^ Additionally, as compared with previously reported Na‐B‐S synthesis strategies, this route achieved low energy consumption, as shown in Figure 1e. Based on the previous preparation methods, high temperatures (>600 °C) were required to activate the reaction between Na_2_S, S, and B within the sealed quartz ampoule under vacuum. Our synthesized temperature could be significantly decreased to 240 °C, as derived from the DSC result (Figure 1a). This significantly decreased synthesized temperature could be rationalized as the auxiliary effect of released H from NaBH_4_. As calculated in Figure 1f, B atoms were more prone to escape from the NaBH_4_ surface (ΔE_B_ = 6.88 eV) than from the B surface (ΔE_B_ = 79.74 eV).

**Figure 2:**
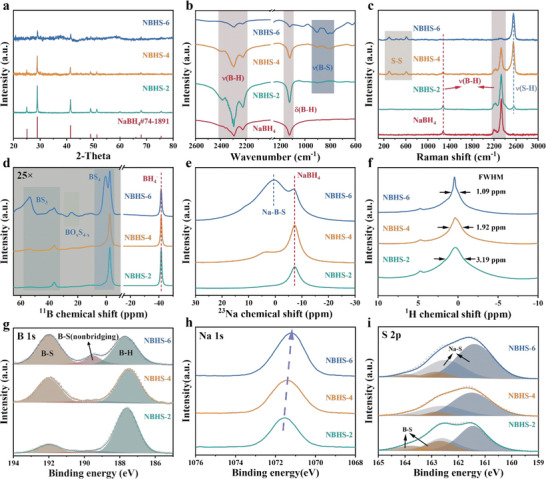
Structure of Na‐B‐H‐S electrolytes. a) XRD patterns, b) FTIR spectra, and c) Raman spectra of the pure NaBH_4_ and NBHS‐x (x = 2, 4, and 6). d) ^11^B NMR, e) ^23^Na NMR, and f) ^1^H NMR spectra of NBHS‐x (x = 2, 4, and 6). g) B 1s, h) Na 1s, and i) S 2p XPS spectra of NBHS‐n (n = 2, 4, and 6).

We mixed and loaded S and NaBH_4_ into the reactor with mass ratios of 2:8, 4:6, 6:4, denoted as NBHS‐2, NBHS‐4, and NBHS‐6, respectively. As shown in **Figure** **2**a, the XRD patterns of the three samples could be assigned to the NaBH_4_ phase (PDF#74‐1891). With increased S loading, NBHS‐6 showed the lowest intensity of the NaBH_4_ pattern, which implied that the degree of the NaBH_4_+S reaction was strongly related to the initial S loading. To evaluate the detailed chemical environment of end products, especially the amorphous Na‐B‐S phase as previously reported, we employed FTIR and Raman spectrums, as compared in Figure 2b,c. According to the FTIR results, typical B‐H stretching vibration bands were located around 2200–2400 cm^−1^, and the B‐H bending mode of ≈1120 cm^−1^ was seen in the NBHS‐2, NBHS‐4, and NBHS‐6 samples, which further verified the residual of NaBH_4_. Among these samples, NBHS‐6 showed the weakest intensity of B‐H bands, which was in accordance with the previous XRD result (Figure 2a). Figure S2 (Supporting Information) shows a detailed comparison between pure NaBH_4_ and NBHS‐6. Regarding pure NaBH_4_, the B‐H bending mode of NBHS‐6 shifted slightly to a lower wavenumber, which could be attributed to the incorporation of S into the residual NaBH_4_ phase.^[^ ^22^ ^]^ New peaks ranging from 800 and 1000 cm^−1^ could be assigned to the B‐S bonds, in which the intensity gradually increased with the S amount. Similar observations could be made in the Raman analysis (Figure 2c). Three fingerprint peaks of NaBH_4_ located at 2330, 2198, and 1278 cm^−1^ gradually decreased from NBHS‐2 to NBHS‐6, which were accompanied by the appearance of new peaks of 2551 cm^−1^, which could be assigned to the S─H bond.^[^ ^26^ ^]^ Moreover, for the NBHS‐4 and NBHS‐6 samples, two obvious new peaks of 295 and 609 cm^−1^ could be indexed to S_6_^−^ and S_7_^−^, respectively, which implied the existence of sodium polysulfide (NaS_x_) in Na‐B‐H‐S.^[^ ^27^ ^]^

We further evaluated the local chemical environment of Na‐B‐H‐S with NMR, as depicted in Figure 2d‐f. For ^11^B NMR spectra (Figure 2d), a resonance peak ≈−41 ppm could be assigned to BH_4_, which was clearly observed in all three samples. Because of their weak intensity, the signals ranging from −10 to 60 ppm were amplified by a factor of 25. All Na‐B‐H‐S samples showed a resonance peak ≈−2.5 ppm, representing B in the BS_4_ tetrahedra with a bridging S environment.^[^ ^28^ ^]^ Among these samples, an additional peak split at 0.49 ppm was observed only in NBHS‐6, which was assigned to the isolated BS_4_ tetrahedra.^[^ ^28^ ^]^ We identified the other two peaks, which were centered at 53 and 36 ppm, as BS_3_. We observed these peaks in all of the Na‐B‐H‐S samples, but the highest intensity was in NBHS‐6. Another peak centered at 24 ppm in the NBHS‐6 sample was assigned to BO_x_S_4‐x_, which may have been introduced during sample preparation.^[^ ^28^ ^]^ Concerning the ^23^Na NMR spectra (Figure 2e), a peak ≈−7 ppm verified the existence of NaBH_4_, while a peak at 0.6 ppm was evident only in the NBHS‐6 sample. This result indicated that S preferentially reacted with BH_4_ before affecting the local environment of Na. According to the ^1^H NMR spectra (Figure 2f), an obvious bump ≈0 ppm could be assigned to the chemical environment change of BH_4_. To quantitatively compare this variation, we used the full width at half maxima (FWHM) to estimate the activity of H, in which the smaller the FWHM value was, the more active the H was.^[^ ^29^ ^]^ The NBHS‐6 sample exhibited the smallest value, which demonstrated that more active H existed in NBHS‐6. This result was attributed to the intensified H─S bonds in NBHS‐6 because more active H could be found in the S─H bond as compared with the B─H bond.

The XPS analysis provided more detailed information, as depicted in Figure g‐i. For the B 1s spectra (Figure 2g), the peak at 187.7 eV was assigned to the B─H bond, in which the intensity decreased as the S mount increased. Two other peaks at 189 and 192 eV were assigned to the isolated BS_4_ and BS_4_ with bridging S, respectively. We noted more obvious thioborates in NBSH‐6, which suggested the possible significant structural differences in NBSH‐6 compared with its counterparts. As displayed in Figure 2h, the Na 1s peaks slightly shifted from 1071.6 to 1071.2 eV with gradually increased S loading, which suggested that the Na^+^ migration became more flexible in NBHS‐6. Concerning S 2p (Figure 2i), the doublet peaks at 163.8 eV (S2p_1/2_) and 162.8 eV (S2p_3/2_) could be assigned to BS_4_ with bridging S, while the doublet peaks at 162.6 eV (S2p_1/2_) and 161.5 eV (S2p_3/2_) could be attributed to terminal S (NaS_x_).^[^ ^30^, ^31^, ^32^ ^]^ With increased S loading, especially for NBHS‐6, the terminal S (NaS_x_) significantly increased, which was in accordance with the previous Raman analysis. The existence of NaS_x_ in NBSH‐6 was supported by the result that a capacity of 100 mAh g^−1^ could be obtained by mixing NBSH‐6 with carbon as the electrode in KB/NBSH‐6||NBSH‐6||Na_15_Sn_4_ (Figure S3, Supporting Information). The morphology of NBHS‐6 is shown in Figures S4 and S5 (Supporting Information). The size of a single particle was at the micrometer scale. Further Energy Dispersive Spectroscopy (EDS) mapping revealed that the Na and S elements were evenly distributed.^[^ ^33^ ^]^

We also found that all of the Na‐B‐H‐S samples included a NaBH_4_ crystal structure and amorphous thioborate (Na‐B‐S). With increased S loading, especially for NBHS‐6, NaBH_4_ and thioborate were tightly bonded to each other with newly formed S─H bonds. Compared with the NBHS‐2 and NBHS‐4 samples, in addition to the higher intensity, NBHS‐6 exhibited a slightly different thioborate structure, with more isolated BS_4_ and NaS_x_. We theoretically evaluated the thermodynamic stability of NaBH_4_‐NaS_x_ and thioborate‐NaS_x_‐NaBH_4_, as shown in Figures S6 and S7 (Supporting Information), respectively. The possibility of spontaneous reactions at various ratios between thioborate‐NaS_x_‐NaBH_4_ interfaces was extremely low, ensuring the stability of the Na‐B‐H‐S electrolyte.

The stability with water or oxygen has been evaluated, as shown in Figure S8 (Supporting Information). The freshly synthesized NBHS‐6 electrolyte was subjected to ambient conditions within a fume hood with a prolonged time. During the initial 1 h, the NBHS‐6 electrolyte exhibited a color transition from off‐white to red (Figure S8a,b, Supporting Information). Additionally, the FTIR (Figure S8c, Supporting Information) comparison revealed that the B─S bonds ranging from 800 and 1000 cm^−1^ disappeared, which could be assigned to the reaction of the NBHS‐6 with water and oxygen. Prolonging the exposure time to 24 h, the NBHS‐6 was deliquescent, and could no longer used as a solid electrolyte (Figure S8d, Supporting Information). This unstable nature with water or oxygen has been reported in most of the hydride‐ and sulfide‐based electrolyte.^[^ ^34^, ^35^ ^]^

**Figure 3:**
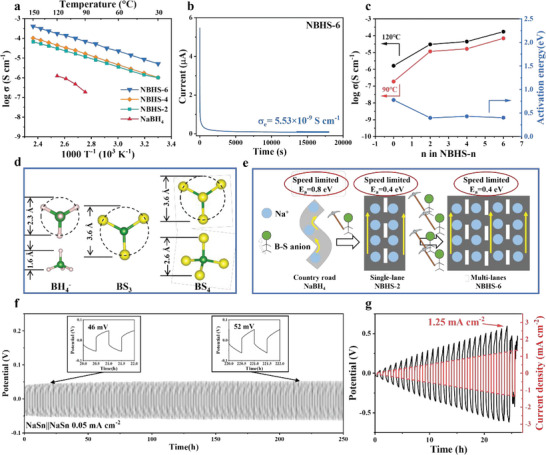
Electrochemical properties of the Na‐B‐H‐S electrolytes. a) Conductivity–temperature curves of pure NaBH_4_ and NBHS‐x (x = 2, 4, and 6) in the temperature range of 30–150 °C. b) Time‐dependent current density curves during polarization at 1 V c) The ionic conductivity at 90 and 120 °C, and the activation energies of NBHS‐x as a function of x. d) Illustrations of the geometric shapes on an identical scale of BH_4_, BS_3_, and BS_4_ anions, BH_4_ and BS_4_ anions are shown in top and side views and BS_3_ is only shown in top view. Green balls: B atoms; pink balls: H atoms; Yellow balls: S atoms. (e) Schematic illustration of the Li‐ion diffusion with S content increasing. Galvanostatic cycling of the Na_15_Sn_4_lNBHS‐6lNa_15_Sn_4_ symmetric cells under (f) constant current density and (g) step‐increased current densities at 125°C.

We evaluated the electrochemical performance of the Na‐B‐H‐S samples, as shown in **Figure** **3**. The ionic conductivity of the Na‐B‐H‐S and NaBH_4_ samples were determined using the EIS, which ranged from 30 to 150 °C, as illustrated in Figure 3a. Compared with pristine NaBH_4_, all of the Na‐B‐H‐S samples exhibited enhanced Na^+^ conductivity. At 90 °C, this value could be increased by three orders of magnitude, and at 120 °C, this value could be increased by at least two orders of magnitude. Among the samples, the NBHS‐6 sample manifested the optimal Na^+^ conductivity, by virtue of more thioborates (BS_4_ and BS_3_) as well as more flexible Na^+^ migration, which was evidenced by its structure characterization. The bulk resistance (*R*_bulk_) and grain boundary resistance (*R*_grain_) were analyzed with EIS, as shown in Figures S9 and S10 (Supporting Information), one semicircle in pristine splits into two semicircles. The higher frequency semicircle represents *R*_bulk_, while the lower frequency semicircle is related to *R*_grain_. The *R*_bulk_ and *R*_grain_ of the NBHS‐6 were reduced to 296 and 373 Ω cm^2^, respectively, at 120 °C. This is attributed to the diminishing interface between NaBH_4_ and amorphous Na‐B‐S within it, as well as the lower impedance in the amorphous Na‐B‐S. In addition, the presence of the amorphous Na‐B‐S phase contributes to good interfacial contact and reduces interfacial impedance.^[^ ^36^ ^]^ A further increase in the S amount and annealing temperature (260 °C) failed to continuously enhance Na^+^ conductivity, as illustrated in Figures S11 and S12 (Supporting Information), respectively. The electronic conductivity of NBHS‐6 was evaluated to 5.53 × 10^−9^ S cm^−1^, which met the basic demand for a solid‐state electrolyte (Figure 3b). We compared the activation energy of Na‐B‐H‐S samples with different NaBH_4_/S mass ratios, as shown in Figure 3c. Interestingly, the activation energy of NaBH_4_ initially decreased to 0.40 in NBHS‐2 and remained relatively stable even within high S loading in the NBHS‐6 sample. With the introduction of thioborates (BS_4_ and BS_3_), the activation barriers could be lowered considerably because of the larger size of thioborates (BS_4_ and BS_3_) than the BH_4_ unit, as shown in Figure 3d. Yu et al.^[^ ^37^ ^]^ indicated that the introduction of a second phase with a large anionic radius could bring significant changes to the local environment of alkali ion in pristine metal hydrides, further facilitating fast ion diffusion. The introduction of a second phase with a large anionic radius is often accompanied by an increase in lattice volume, which could augment the rotational freedom of the anions.^[^ ^38^ ^]^ The size of BS_4_/BS_3_ was larger than the BH_4_ unit as shown in Figure 3d. The larger BS_4_/BS_3_ anion embedded in the anionic frameworks may contribute to the reconstruction of wider Na^+^ diffusion channels, which result in reduced electrostatic interactions between the Na^+^ and the anionic skeleton, thus facilitating faster Na^+^ mobility. A rational explanation was provided for NBHS‐6, in which although the ionic conductivity increased, the activation energy was relatively stable without obvious change (Figure 3e). The Na^+^ conductivity of initial NaBH_4_ was rather low, which was like Na^+^ walking on a country road with limited speed, which was equivalent to active energy barrier (*E*_a_). In the case of NBHS‐2 and NBHS‐6, the interaction between BH_4_ anions was reduced while also broadening the transportation space for Na^+^, like the construction team (BS_4_ and BS_3_) widened the original country road and turned it into a highway, further dramatically increasing the moving speed. With the further accumulation of BS_4_/BS_3_ in NBHS‐6, the speed limit of the highway, which was similar to *E*_a_, no longer changed; however, BS_4_/BS_3_ turned from single‐lane to multi‐lanes to ensure greater Na^+^ transportation, as reflected by the enhanced Na^+^ conductivity.

The synthesized NBHS‐6 electrolyte exhibited a Na^+^ conductivity of 1.7 × 10^−4^ S cm^−1^ at 120 °C. Given the low melting point of Na metal (97 °C), the Na_15_Sn_4_ alloy was employed to assemble devices (Figure S13, Supporting Information). Figure 3f shows the constant current cycling of the NBHS‐6 electrolyte in a symmetrical cell with Na_15_Sn_4_ electrodes at 125 °C. It is demonstrated that NBHS‐6 electrolyte can reversibly transfer Na^+^ for 250 h. Detailed information has been magnified and is shown in the inset in Figure 3f, in which the overpotential for the initial 20 h was 46 mV, and a value of 52 mV was obtained after 200 h, which demonstrated the stability and compatibility between NBHS‐6 electrolyte and Na_15_Sn_4_ anode. The smaller overpotential variation indicated that no significant side reactions occurred with the critical current density of 1.25 mA cm^−2^ (Figure 3g).

We conducted a CV test on S||Na_15_Sn_4_ device within the voltage range of 0.1–5 V at a scanning rate of 0.5 mV s^−1^. In the CV curve (Figure S14, Supporting Information), a broad oxidation peak is observed at 1.8 V, accompanied by a corresponding reduction peak positioned at 1 V. These peaks are attributed to the reversible conversion between S and Na_2_S, respectively. No other oxidation peak was observed, indicating the stability of the NBHS‐6 electrolyte up to 5 V.

**Figure 4:**
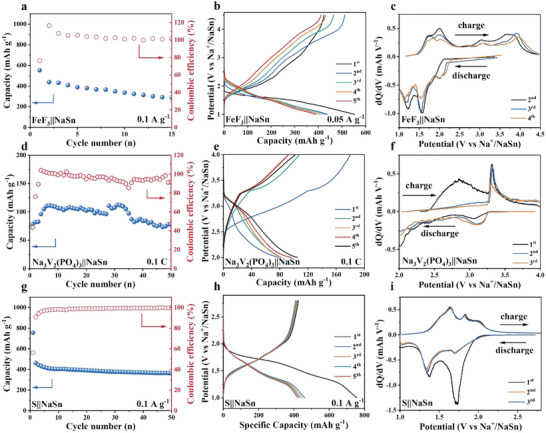
Performance of All‐solid‐state sodium‐ion batteries with NBHS60 at 125 °C. a) Cycling performance of FeF_3_||Na_15_Sn_4_ cell at 0.05 A g^−1^ and the b) corresponding GDC curves. c) Differential capacity curves (dQ/dV) of the FeF_3_ electrode were obtained from the galvanostatic discharge–charge curves. d) Cycling performance of Na_3_V_2_(PO_4_)_3_||Na_15_Sn_4_ cell at 0.1 C and the e) corresponding GDC curves. f) Differential capacity curves (dQ/dV) of the Na_3_V_2_(PO_4_)_3_ electrode obtained from the galvanostatic discharge‐charge curves. g) Cycling performance of S||Na_15_Sn_4_ cell at 0. 1 A g^−1^ and the h) corresponding GDC curves. i) Differential capacity curves (dQ/dV) of the S electrode obtained from the galvanostatic discharge–charge curves.

To evaluate the universality of the NBHS‐6 electrolyte, we used various cathode materials, including FeF_3_ (1.0–4.5 V), Na_3_V_2_(PO_4_)_3_ (2.0–4.0 V), and S (1.2–2.8 V), as shown in **Figure** **4**. The FeF_3_||Na_15_Sn_4_ device exhibited a reversible capacity of 290 mAh g^−1^ at 50 mA g^−1^ after 15 cycles (Figure 4a). It should be noted here the cycling stability of FeF_3_ cathode is still unfavorable, due to multiphase redox steps involved in metal fluorides, with extensive structural rearrangements.^[^ ^39^, ^40^ ^]^ Figure 4b shows the corresponding galvanostatic charge–discharge (GDC) curves for the different cycles. Figure 4c shows the differential capacity versus voltage (dQ/dV) curves within the voltage window of 1.0–4.5 V. The dQ/dV curves revealed that the oxidation/reduction peaks were slightly lower than the theoretical reaction potential between FeF_3_ and Na^+^, which further confirmed that the NBHS‐6 electrolyte participated in the reaction. The oxidation peaks located between 2.5 and 4.5 V and the reduction peak at 2 V were consistent with Na^+^ insertion/extraction reaction. The oxidation peaks located at 2 and 1.7 V and the reduction peaks at 1.6 and 1.2 V, however, were consistent with the conversion reaction between NaFeF_3_ and sodium fluoride (NaF).^[^ ^41^ ^]^

To evaluate the compatibility of NBHS‐6 electrolyte for commercial electrode, we prepared the Na_3_V_2_(PO_4_)_3_||Na_15_Sn_4_ device. As illustrated in Figure 4d, the Na_3_V_2_(PO_4_)_3_||Na_15_Sn_4_ showed an exciting capacity of 78 mAh g^−1^ at a current density of 0.1 C after 50 cycles. The corresponding GDC curves (Figure 4e) demonstrated that a large deviation for the first discharge process could generate a stable interface between the NBHS‐6 electrolyte and Na_3_V_2_(PO_4_)_3_ cathode. From the second cycle onward, the GDC curves overlapped, which indicated the reversibility of the Na_3_V_2_(PO_4_)_3_||Na_15_Sn_4_ device. Figure 4f shows the dQ/dV curves within the voltage window of 2.0–4.0 V. The oxidation peak located at 3.3 and the reduction peak at 3.2 V were consistent with Na_3_V_2_(PO_4_)_3_||Na_15_Sn_4_.^[^ ^42^ ^]^ The oxidation peak located at 2.8 and the reduction peak at 2.1 V were consistent with Na_2_S_x_ in NBHS‐6.

When assembled with the S cathode and Na_15_Sn_4_ anode, the S||Na_15_Sn_4_ device could achieve a capacity of 364 mAh g^−1^ after 50 cycles, as shown in Figure 4g. Apart from the first discharge curve, the GDC curves of the S||Na_15_Sn_4_ device overlapped from the second cycle onward. Figure 4i shows the dQ/dV curves within the voltage window of 1.0–2.6 V. The dQ/dV curves revealed two oxidation peaks located at 1.6 and 1.8 V and two reduction peaks at 1.3 and 1.7 V. The oxidation peak located at 1.6 V and the reduction peak at 1.3 V was consistent with the reversible conversion between Na_2_S_4_ and Na_2_S, whereas the oxidation peak located at 1.8 V and the reduction peak at 1.7 V were consistent with the reversible conversion between S and Na_2_S_4_. We further investigated the rate performance of the S||Na_15_Sn_4_ at different current densities (Figure S15, Supporting Information). At current densities of 0.05 0.1, 0.2, 0.4, and 0.8 A g^−1^, the average discharge capacities of the S||Na_15_Sn_4_ electrode were 1003, 507, 381, 272, and 152 mAh g^−1^, respectively. The capacity could recover to 331 mAh g^−1^ when the current density returned to 0.2 A g^−1^. The corresponding GDC curves at various current densities are illustrated in Figure S16 (Supporting Information). Even at a high current density of 0.8 g^−1^, the voltage plateau was well preserved. Once assembled with the adapted processes, these three typical cathode materials, Na‐free cathode (S and FeF_3_), high‐voltage (Na_3_V_2_(PO_4_)_3_ and FeF_3_), and high‐capacity (FeF_3_ and S) materials, were all compatible with the NBHS‐6 electrolyte.

## 3 Conclusion

In conclusion, we proposed a one‐step dehydrogenation‐assisted strategy to synthesize the novel thio‐borohydride Na‐B‐H‐S (NaBH_4_/Na‐B‐S) electrolyte. By using NaBH_4_ instead of B as a starting material, both raw material cost and preparation temperature were significantly reduced. Theoretical modeling demonstrated that B atoms were readily released from NaBH_4_ with much less energy of 6.88 eV than 79.74 eV of elemental B. The synthesized Na‐B‐H‐S (NaBH_4_/Na‐B‐S) electrolyte could exhibit a Na^+^ conductivity of 1.7 × 10^−4^ S cm^−1^ at 120 °C, which was an increase of three orders of magnitude compared with pristine NaBH_4_, by virtue of larger thioborates (BS_4_ and BS_3_) as well as more flexible Na^+^ migration. When assembled with the S cathode and Na_15_Sn_4_ anode, the S||Na_15_Sn_4_ device could manifest a capacity of 364 mAh g^−1^ after 50 cycles. Excitingly, the Na_3_V_2_(PO_4_)_3_||Na_15_Sn_4_ and FeF_3_||Na_15_Sn_4_ devices achieved a promising performance that demonstrated the potential for further commercialization.

## 4 Experimental Section

### Material Synthesis

The raw materials applied for the preparation of Na‐B‐H‐S solid electrolytes (SEs) were NaBH_4_ (98% purity, Aladdin, Shanghai, China) and S (99.5% purity, Sinopharm, Shanghai, China). For the synthesis of NBHS SEs, NaBH_4_ and S is added to argon (Ar)‐filled agate tank and then ball‐milled the mixture at 400 rpm for 4 h in the mass ratio of 8:2, 6:4, and 4:6. The ratio of grinding media to powder was 10:1. It then placed the mixture in a glass bottle, heated it to 240 °C at the heating rate of 1 °C min^−1^ with a muffle furnace (KSL‐1100X‐S, Hefei Kejing, Anhui, China) under an Ar atmosphere, and held for 1 h. After cooling to room temperature, the SEs were ground using mortar. Na_15_Sn_4_ alloy was synthesized by heating the mixture of tin powder and sodium foil in a stoichiometric ratio at 150 °C with mechanical stirring at 200 rpm for three days under an argon atmosphere. All synthesis procedures were conducted inside an Ar‐filled glovebox (O_2_ < 0.01 ppm; H_2_O < 0.01 ppm).

### Characterization Methods

It performed differential scanning calorimetry (DSC) tests using a TGA‐DSC, Netzsch, STA 409 PC (Selb, Germany). It conducted X‐ray diffraction (XRD) analysis on a Bruker D8 Advance X‐ray diffractometer (Karlsruhe, Germany) with Cu K*α* radiation (*λ* = 1.5406 Å). It used a field‐emission scanning electron microscope (TESCAN VEGA 3 XMU, Brno, Czech Republic) equipped with energy‐dispersive X‐ray photoelectron spectroscopy (EDS) to analyze the sample morphology. It recorded the Fourier‐transformed infrared (FTIR) spectra of the SEs on an infrared spectrometer (NICOLET iS50 FT‐IR, ThermoFisher Scientific, Waltham, MA, USA). It recorded the Raman spectra of the SEs on an Integrated FTIR‐Raman spectrometer (Bruker). It performed the ^23^Na, ^11^B, and ^1^H nuclear magnetic resonance (NMR) tests on a Bruker NEO 400 m solid‐state NMR spectrometer and conducted S 2p, Na 1s, and B 1s XPS spectra on an ESCALAB 250Xi spectrometer (ThermoFisher Scientific). All samples were prepared in a glove box.

### Density Functional Theory Calculations

Theoretical calculations based on density functional theory (DFT) were performed using a spin‐polarized generalized‐gradient approximation with a PW91 functional and a double numerical basis set, including a polarization function as implemented in the DMol3 package. The Brillouin zone was sampled by 2 × 2 × 1 special k‐points using the Monkhorst‐Pack scheme. The (01−1) surfaces of pure B and (110) surface of NaBH_4_ crystals were represented by 3‐D slab models in a periodic boundary condition. The slab models were constructed initially as 2 × 3 × 1 super cells for B and 3 × 3 × 4 super cells for NaBH_4_, respectively. The exposed surface was given a vacuum layer of >10 Å to avoid interactions with its periodic image in the c direction. The energy (Δ*E*_B_) required to remove one B atom from the surfaces is defined as

(1) $${\Delta E}_{B}=E_{\mathrm{total}}(N_{B}-1)+E_{B}-E_{\mathrm{total}}\left(N_{B}\right)$$

where *E*_total_ (*N*_B_−1) is the total energy of the surface model with the (*N*_B_−1) number of B atoms, *E*_B_ is the energy of one isolated B atom, and *E*_total_ (*N*_B_) is the total energy of the surface model with the *N*_B_ number of B atoms.

### Electrochemical Measurements

It determined the bulk resistance of the samples from electrochemical impedance spectroscopy (EIS) using a High‐Frequency Impedance Test System (HT‐Z2‐HF, TOYO Corporation, Tokyo, Japan). It completed the measurements at a temperature ranging from 30 to 150 °C. The powder was pelletized in a 10 mm diameter at room temperature under the pressure of 200 MPa.

It measured electronic conductivity via DC polarization using a 13 mm diameter Polyetheretherketone (PEEK) mold, in which powder was pressed between two stainless steel pistons. It applied a voltage of 1 V for 5 h on an electrochemical workstation (Gamry INTERFACE1010E, Warminster, PA, USA).

It pressed NBHS‐6 SE (100 mg) in a 13 mm diameter PEEK mold to form a pellet. The powder Na_x_Sn (20 mg) was uniformly spread on either side and kept in contact with the SE. The cell was then sandwiched between two stainless steel rods and held under pressure using a custom‐made cell. It performed galvanostatic cycling of the NBHS‐6 cells at 0.05 mA cm^−2^. It tested step‐increased current densities from 0.05 to 1.35 mA cm^−2^.

For the fabrication of Na_15_Sn_4_|NBHS‐6|Na_15_Sn_4_ symmetric cells, ≈100 mg NBHS‐6 powder was pressed into a 13 mm pellet at 200 MPa for 1 min, and then 10 mg Na_15_Sn_4_ powder was added to both sides of the pellet and pressed at 200 MPa for another 1 min. The S–graphene oxide (GO) composite was prepared by mixing the S with GO (sheet diameter: >5 um, number of layers: 1–6 layers, XFNANO) at a weight ratio of 3:7. The Na_3_V_2_(PO_4_)_3_/NBHS‐6/graphene composite was prepared by mixing the Na_3_V_2_(PO_4_)_3_ (20 µm, Shenzhen Kejing), NBHS‐6 with graphene (sheet diameter: 0.5–5 µm, thickness: 0.8 nm, monolayer rate: 80%, XFNANO) at a weight ratio of 4:3:3. The FeF_3_/NBHS‐6/ Ketjen black (KB) composite was prepared by mixing the FeF_3_, NBHS‐6 with KB at a weight ratio of 7:3:10. First, 100 mg NBHS‐6 powder was placed into a PEEK cylinder and pressed at 200 MPa for 1 min (13 mm diameter). On one side of the NBHS‐6 pellet, the cathode composite was spread. Finally, 20 mg Na_15_Sn_4_ powder was spread over the other side of the NBHS‐6 pellet. The S||Na_15_Sn_4_ cell was cycled at a current density of 100 mA g^−1^ and voltage range between 1.0–2.8 V versus Na_15_Sn_4_. The FeF_3_||Na_15_Sn_4_ cell was cycled at a current density of 50 mA g^−1^ and voltage range between 1.0–4.5 V versus Na_15_Sn_4_. The Na_3_V_2_(PO_4_)_3_||Na_15_Sn_4_ cell was cycled at a current density of 11.8 mA g^−1^ and voltage range between 2.0–4.0 V versus Na_15_Sn_4_. All cells were maintained at 125 °C for 24 h and then used for electrochemical tests. It assembled all cells in an Ar‐filled glove box with O_2_ and H_2_O < 0.01 ppm.

## Conflict of Interest

The authors declare no conflict of interest.

## Supporting information

Supporting Information

## Data Availability

The data that support the findings of this study are available from the corresponding author upon reasonable request.
