# Supplementary material for: Low‐Cost Preparation of High‐Performance Na‐B‐H‐S Electrolyte for All‐Solid‐State Sodium‐Ion Batteries
Source: Adv Sci (Weinh). 2023 Sep 25;10(32):2302618. doi: 10.1002/advs.202302618 (PMC10646275; doi:10.1002/advs.202302618)
Supplement: Supplementary file 1 — Supporting Information [file ADVS-10-2302618-s001.pdf]

ADVANCED SCIENCE
Open Access

Supporting Information

for *Adv. Sci.*, DOI 10.1002/advs.202302618

Low-Cost Preparation of High-Performance Na-B-H-S Electrolyte for All-Solid-State Sodium-Ion Batteries

Wei Zhou, Changsheng Song, Shuyang Li, Miao Liu, Huiwen He, Shaoyu Yang, Jin Xie, Fei Wang, Fang Fang, Dalin Sun, Jie Zhao and Yun Song**

Supplementary Information

Low-cost preparation of high-performance Na-B-H-S electrolyte for all-solid-state sodium-ion batteries

Wei Zhou[1#], Changsheng Song[1,2#], Shuyang Li[1], Miao Liu[3], Huiwen He[4], Shaoyu Yang[5], Jin Xie[5], Fei Wang[1], Fang Fang[1], Dalin Sun[1], Jie Zhao[1,2*] and Yun Song[1*]

[1] *Department of Materials Science, Fudan University, Shanghai 200433, China.*

[2] *State Key Laboratory of Molecular Engineering of Polymers, Department of Material Science, Fudan University, Shanghai 200438, China*

[3] *Beijing National Laboratory for Condensed Matter Physics and Institute of Physics, Chinese Academy of Sciences, Beijing 100190, China.*

[4] *State Key Laboratory of Power Grid Environmental Protection, China Electric Power Research Institute, Wuhan 430074, China.*

[5] *School of Physical Science and Technology, ShanghaiTech University, Shanghai 201210, China.*

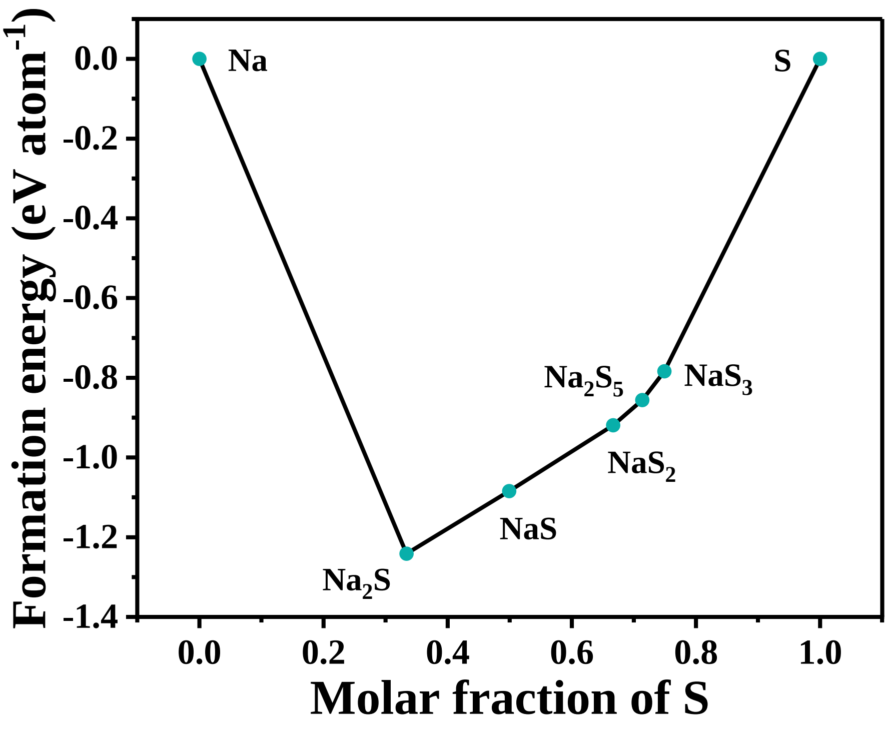

Figure S1. The formation energy of Na and S, depending on the molar fraction of S.

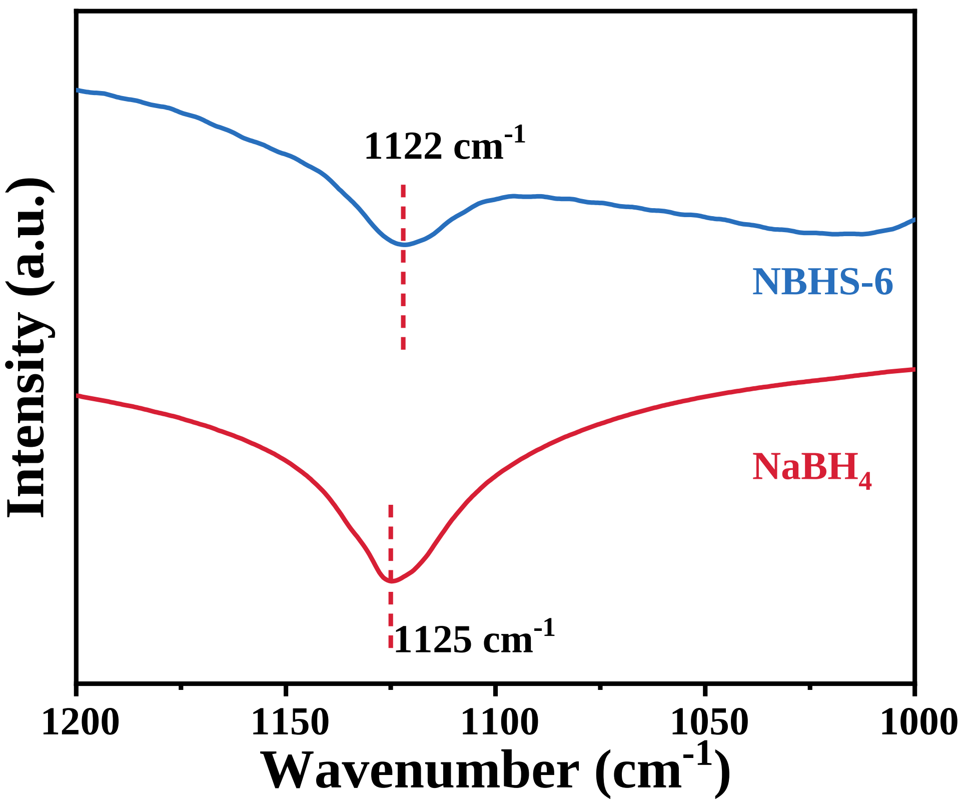

Figure S2. The detailed enlarged picture of pure $NaBH_4$ and NBHS-6 in Fig. 2b.

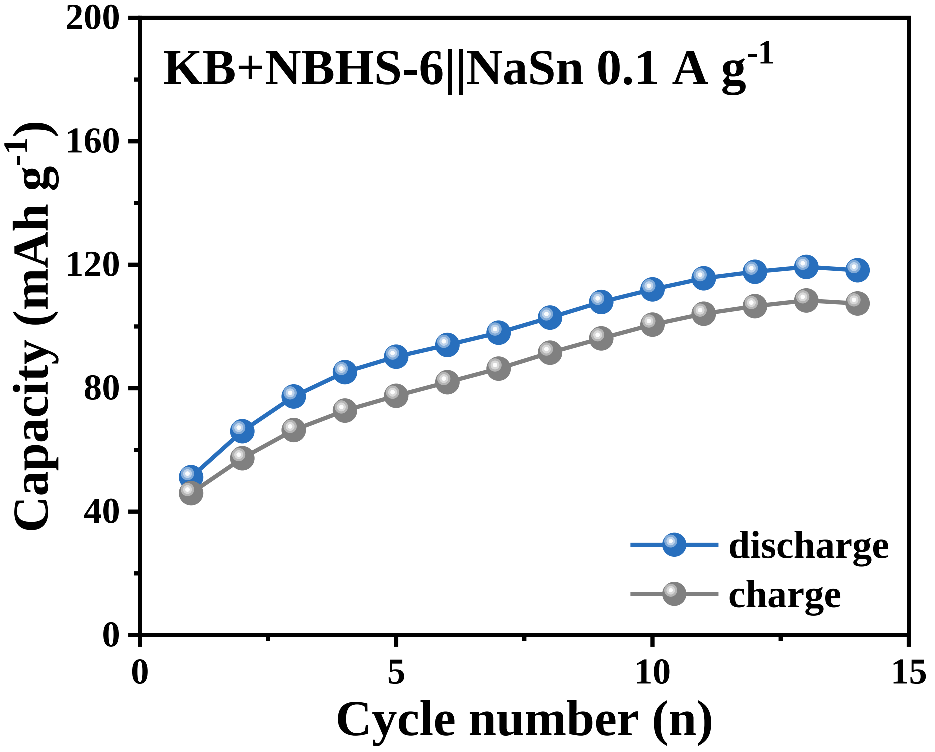

Figure S3. The cycling performances of KB+NBHS-6||NaSn at 0.1 A g^{-1}.

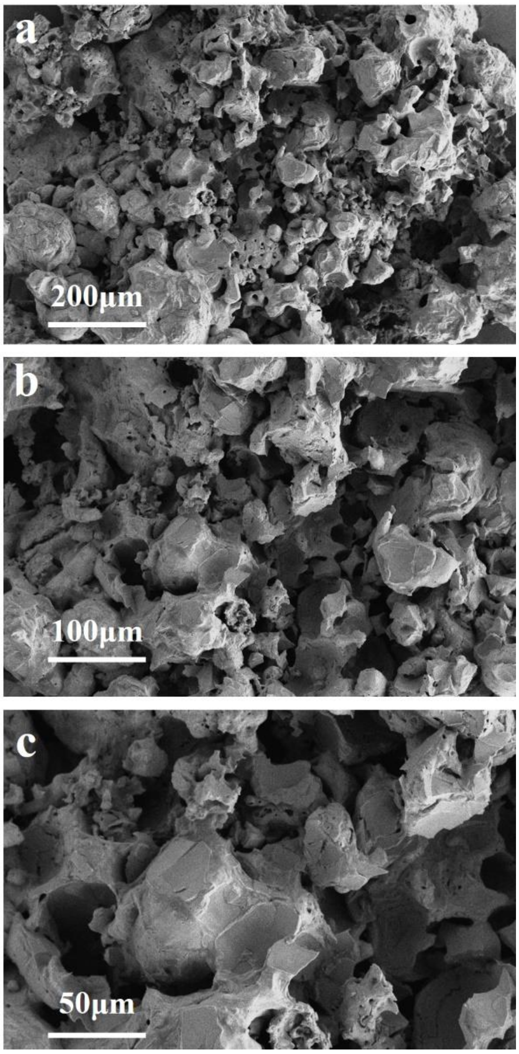

Figure S4. SEM image of NBHS-6 electrolyte at (a) 250x, (b) 500x, (c) 1000x.

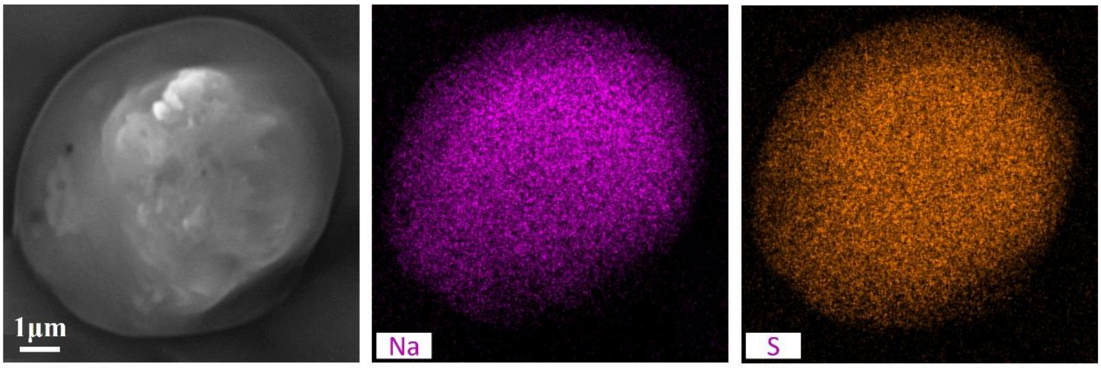

Figure S5. EDS mapping of NBHS-6 electrolyte with the scanned elements Na and S.

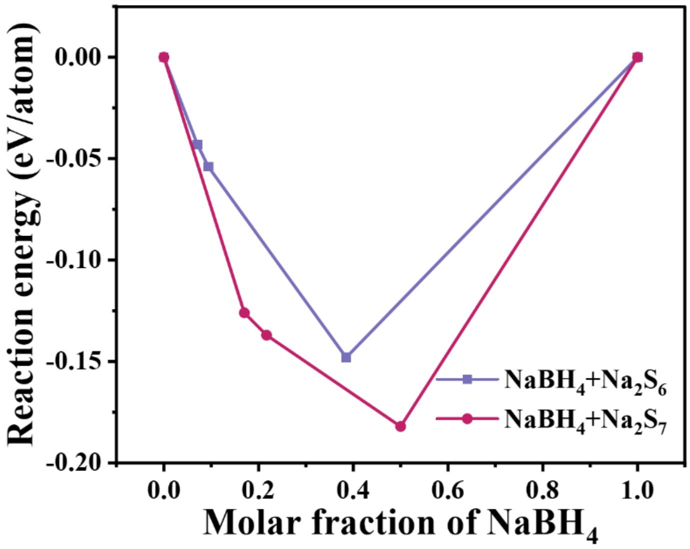

Figure S6. The mutual reaction energies of $NaBH_4$ and NaS_x.

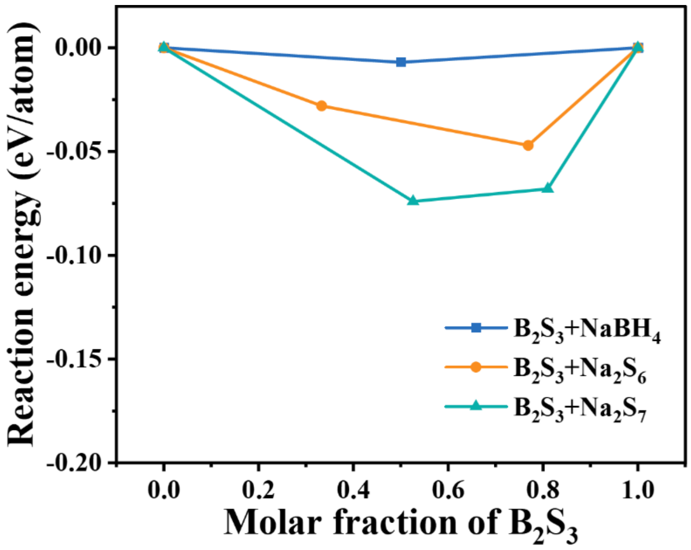

Figure S7. The mutual reaction energies of B_2S_3-NaS_x-$NaBH_4$.

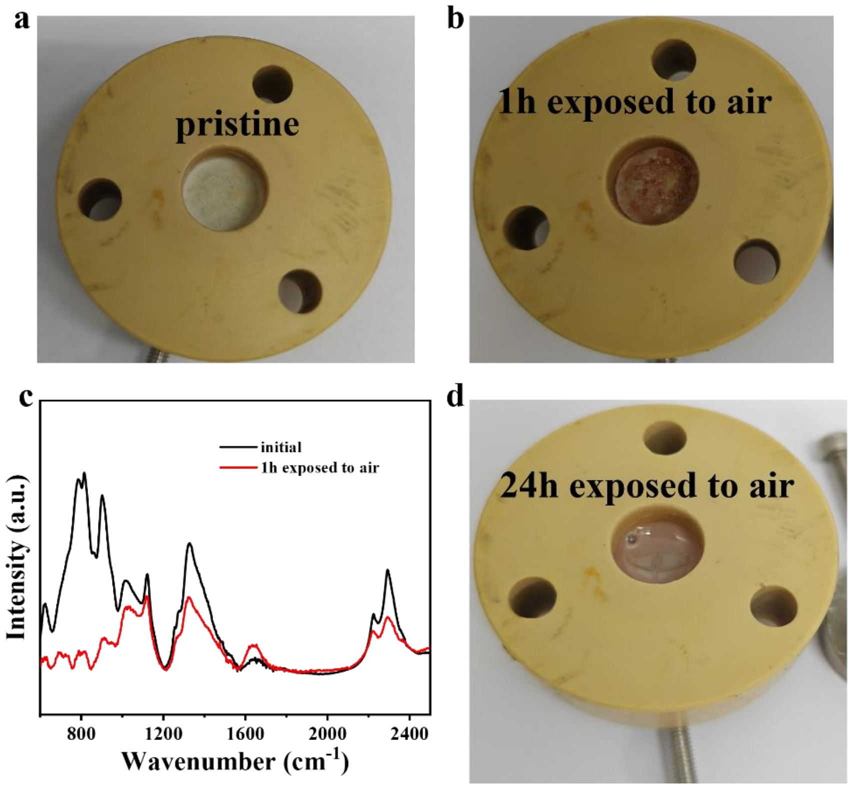

Figure S8. Stability of the NBHS-6 electrolyte with air. (a) pristine NBHS-6. (b) NBHS-6 after 1h exposure to air. (c) FTIR spectra of pristine NBHS-6 and NBHS-6 after 1h exposure to air. (d) NBHS-6 after 24h exposure to air.

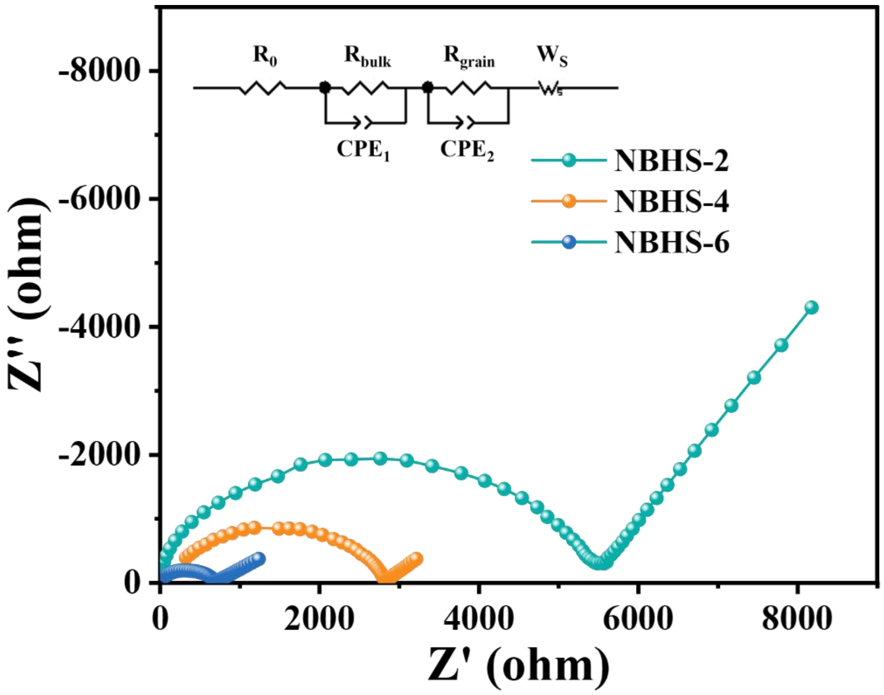

Figure S9. EIS plots of NBHS-2, 4 and 6 at 120°C.

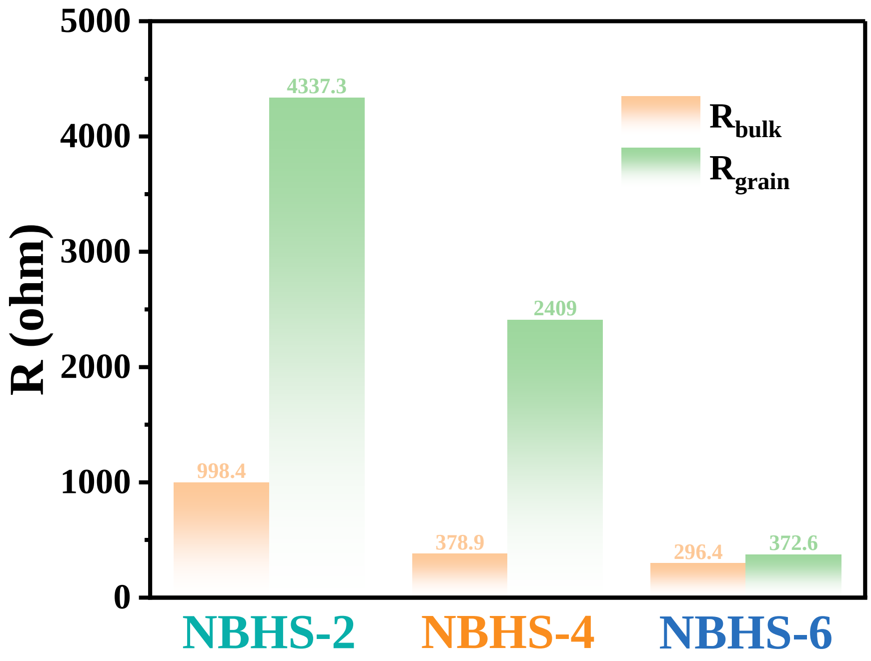

Figure S10. R_{bulk} and R_{grain} of NBHS-2, 4 and 6 at 120°C.

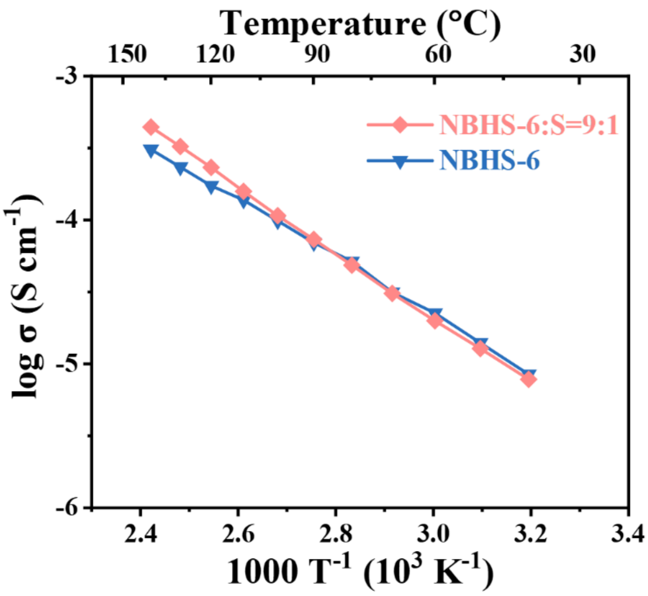

Figure S11. Conductivity–temperature curves of NBHS-6 and NBHS-6:S=9:1 in the temperature range of 40-140°C.

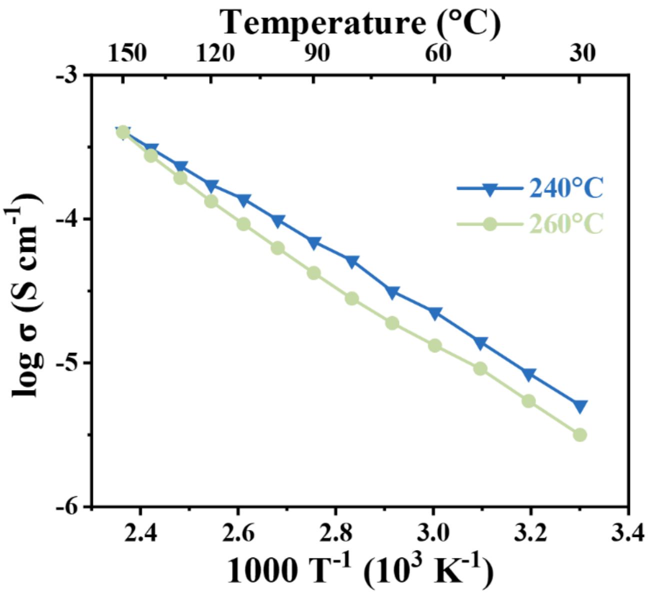

Figure S12. Comparation of the Conductivity–temperature curves of NBHS-6 synthesized at 240 and 260°C, respectively, in the temperature range of 40-140°C.

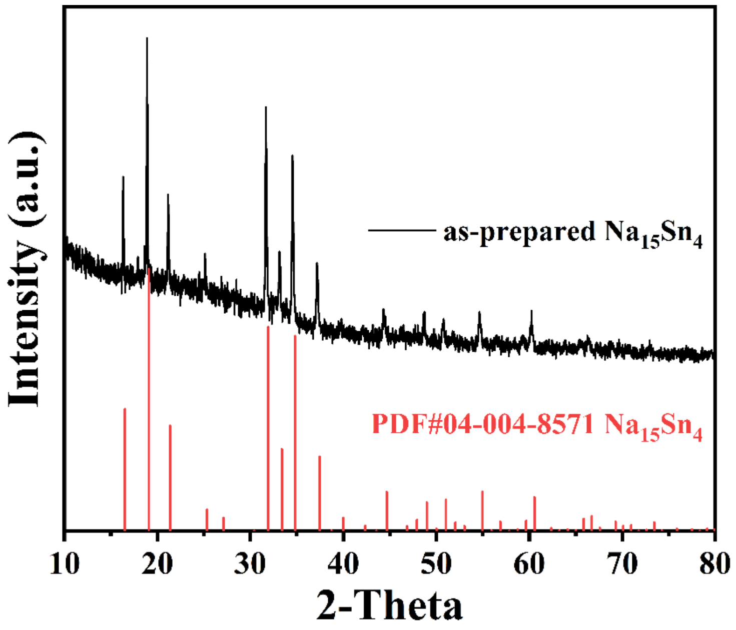

Figure S13. XRD pattern of as-prepared $Na_{15}Sn_4$.

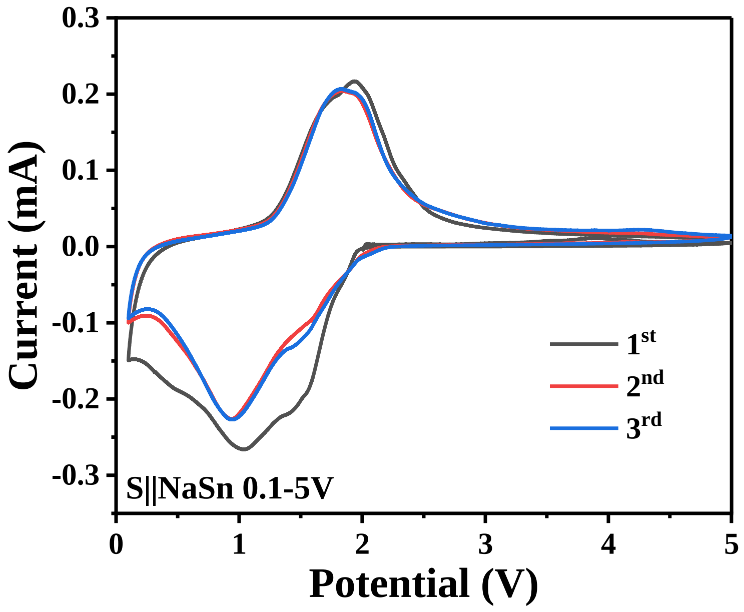

Figure S14. CV curve of S|NBHS-6|$Na_{15}Sn_4$.

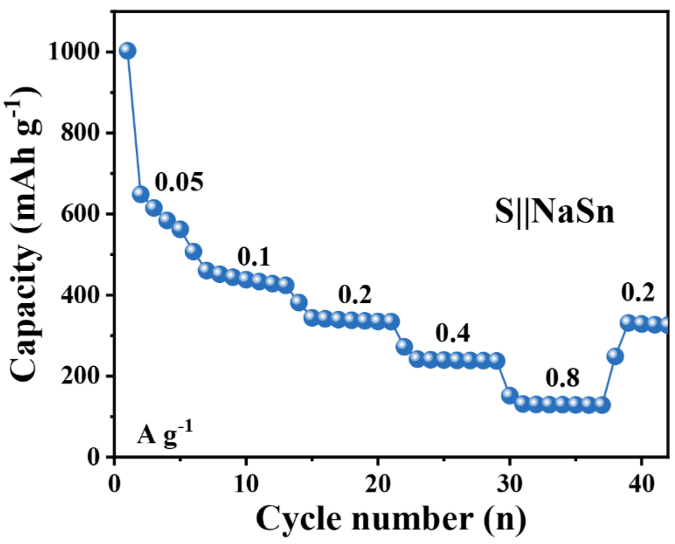

Figure S15. Rate performances of the S electrode at different current densities between 0.05 and 0.8 A g^{-1}.

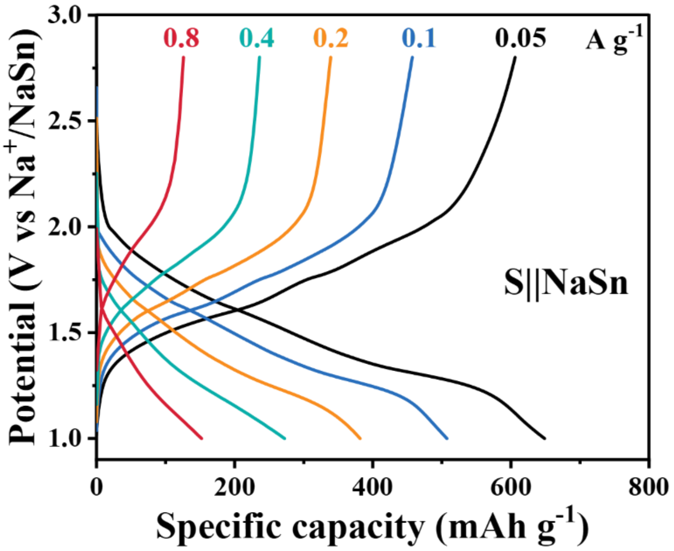

Figure S16. The corresponding GDC curves of S electrode at various current densities between 0.05 and 0.8 A g^{-1}.
